# Effect of a Titanium Tetrafluoride Varnish in the Prevention and Treatment of Carious Lesions in the Permanent Teeth of Children Living in a Fluoridated Region: Protocol for a Randomized Controlled Trial

**DOI:** 10.2196/resprot.9376

**Published:** 2018-01-26

**Authors:** Beatriz Martines Souza, Daiana Moreli Soares Santos, Aline Silva Braga, Natália Mello Dos Santos, Daniela Rios, Marilia Afonso Rabelo Buzalaf, Ana Carolina Magalhães

**Affiliations:** ^1^ Department of Biological Sciences Bauru School of Dentistry University of São Paulo Bauru Brazil; ^2^ Department of Pediatric Dentistry, Orthodontic and Public Health Bauru School of Dentistry University of São Paulo Bauru Brazil

**Keywords:** clinical trial, dental caries, topical fluorides

## Abstract

**Background:**

Titanium tetrafluoride (TiF_4_) has regained interest due to new formulations that have been shown to be more effective against tooth demineralization than sodium fluoride (NaF) formulations in vitro and in situ.

**Objective:**

The aim of this study is to evaluate the effect of two types of varnishes (4% TiF_4_ and a commercial 5% NaF) on the prevention of carious lesions and the treatment of noncavitated enamel carious lesions in the permanent teeth of children living in a fluoridated area.

**Methods:**

This randomized, controlled, parallel and single-blind clinical trial involves 63 children, 6-7 years old, living in Bauru, São Paulo, Brazil. Children were selected according to their caries activity (ie, presence of at least 1 tooth with a Nyvad score of 1) and randomly divided into the following treatment categories: 4% TiF_4_ varnish (2.45 % F^-^, pH 1, FGM); 5% NaF varnish (2.26% F^-^, pH 5, Duraphat, Colgate) and control (placebo varnish, pH 5, FGM). The varnishes will be applied on all permanent teeth, once a week for 4 weeks and they will be reapplied only once 6 and 12 months after the study begins. Two calibrated examiners will carry out the clinical examination (International Caries Detection and Assessment System [ICDAS] and Nyvad indexes, kappa>.8) at baseline, before the first application, after the 1st, 6th, 12th, and 18th month of the study begins. Furthermore, quantitative fluorescence changes will be measured using Quantitative Light-Induced Fluorescence (QLF). The degree of patient satisfaction with the treatment will also be computed. The data will undergo statistical analysis (*P*<.05).

**Results:**

This ongoing study is funded by funding agencies from Brazil (São Paulo Research Foundation, FAPESP-015/14149-1, and National Council for Scientific and Technological Development, CNPq-401313/2016-6). We expect to confirm the efficacy of TiF_4_ on the prevention and treatment of carious lesions by comparing it to NaF varnish. The subjects are under 1 month evaluation and the dropout was about 8%. No differences between the treatments have been detected at the first month so far (*P*>.05).

**Conclusions:**

If our hypothesis is confirmed, TiF_4_ varnish can be marketed and applied at the individual level and used in community programs to control dental caries.

**Trial Registration:**

Brazilian Clinical Trials Registry: RBR-5VWJ4Y; http://www.ensaiosclinicos.gov.br/rg/?q=RBR-5VWJ4Y (Archived by WebCite at http://www.webcitation.org/6wUurEnm7)

## Introduction

Fluoride varnishes are a feasible approach for preventing and treating carious lesions at the individual level and in public health programs, due to its good cost-benefit compared to initial carious lesions restorations, when they eventually progress to cavitation and have a significant negative impact in quality of life [1,2].

Due to the polarization of caries [3] and inequality in health services access, treatment is available to only a small portion of the population [3,4]. This fact requires the attention of authorities and appropriate public health interventions [4,5]. Based on this new panorama of the disease, researchers have sought to improve the effect of conventional fluorides or alternatively to test nonconventional fluorides (eg, fluorides [F] containing polyvalent metals, such as stannous fluoride [SnF_2_] and titanium tetrafluoride [TiF_4_]) [6,7].

Several in vitro and in situ studies have shown that an experimental 4% TiF_4_ is more effective than NaF at reducing demineralization and improving remineralization [7-9]. The titanium ions from TiF_4_ react with dental apatite, forming an acid resistant, glaze-like layer that is rich in hydrated titanium phosphate and titanium dioxide [10]. Furthermore, TiF_4_ varnish induces a higher deposition of calcium fluoride (CaF_2_) than NaF varnish on both intact and demineralized enamel surface [10].

Recent in situ study demonstrated that 4% TiF_4_ varnish was the only treatment capable of improving enamel remineralization regardless of cariogenic activity, while NaF varnish failed in preventing further demineralization under high cariogenic activity [7]. This result supports the hypothesis of the present study that TiF_4_ varnish could be more effective than NaF varnish in preventing and treating carious lesions in the permanent teeth of children living in a fluoridated area.

The aim of this clinical protocol is to evaluate the effect of 4% TiF_4_ varnish compared to a commercial 5% NaF varnish on the prevention of carious lesions and the treatment of noncavitated enamel carious lesions in the permanent teeth of children living in a fluoridated area.

## Methods

### Ethical Aspects

The protocol of this study was submitted and approved by the local ethics committee (Number: 59787116.2.0000.5417, Ethics Committee of the Bauru School of Dentistry, University of São Paulo, Brazil) and by the registration of clinical research in the Brazilian Clinical Trials Registry (Number: RBR-5VWJ4Y). The research protocol was also approved by the Municipal Secretariat for Education of Bauru (São Paulo, Brazil) and by the 5 municipal schools (see Table 1) enrolled in the study. Thereafter, the parents and/or guardians responsible for the study participants (eg, children aged 6-7 years) received and signed an informed consent form prior to their involvement in the research. The children also received a consent form, with age-appropriate language, explaining how the research would be conducted. The children were free to accept or reject participation in the study. Upon receiving approval and consent from all involved parties, the study began.

### Study Design

This is a randomized, controlled, parallel, single-blind, and three-armed (ie, 4% TiF_4_ varnish, 5% NaF varnish and placebo varnish) clinical trial with a duration of 18 months. It involves 63 children (37 males and 26 females) between 6-7 years of age, coming from public schools of Bauru city, an area that is optimally fluoridated. An experimental number of 20 children per treatment group was previously calculated considering an α error of 5%, β error of 20%, a dropout rate of 30%, and a caries incidence rate after a period of 2 years of 15% for fluoride group and 42% for control group [11].

Children were selected according to their caries activity (ie, at least 1 active white spot lesion present on the smooth surface of their permanent dentition with a score of 1 according to the Nyvad index [12]) and randomly allocated to one of the 3 treatment options ensuring stratified block randomization into each group: 4% TiF_4_ varnish (2.45% F^-^, pH 1, FGM); 5% NaF varnish (2.26% F^-^, pH 5, Duraphat, Colgate) and control (placebo varnish, pH 5, FGM).

**Table 1:** Distribution of selected schools according to the regions of Bauru city (São Paulo, Brazil).

Region	Name of school
North	EMEF^a^ José Romão
	EMEF Geraldo Arone
South	EMEF Santa Maria
East	EMEF Thereza Tarzia - Irmã Rosamaria Tarzia
West	EMEF Ivan Engler de Almeida

^a^EMEF: municipal school of fundamental education.

**Figure 1:**
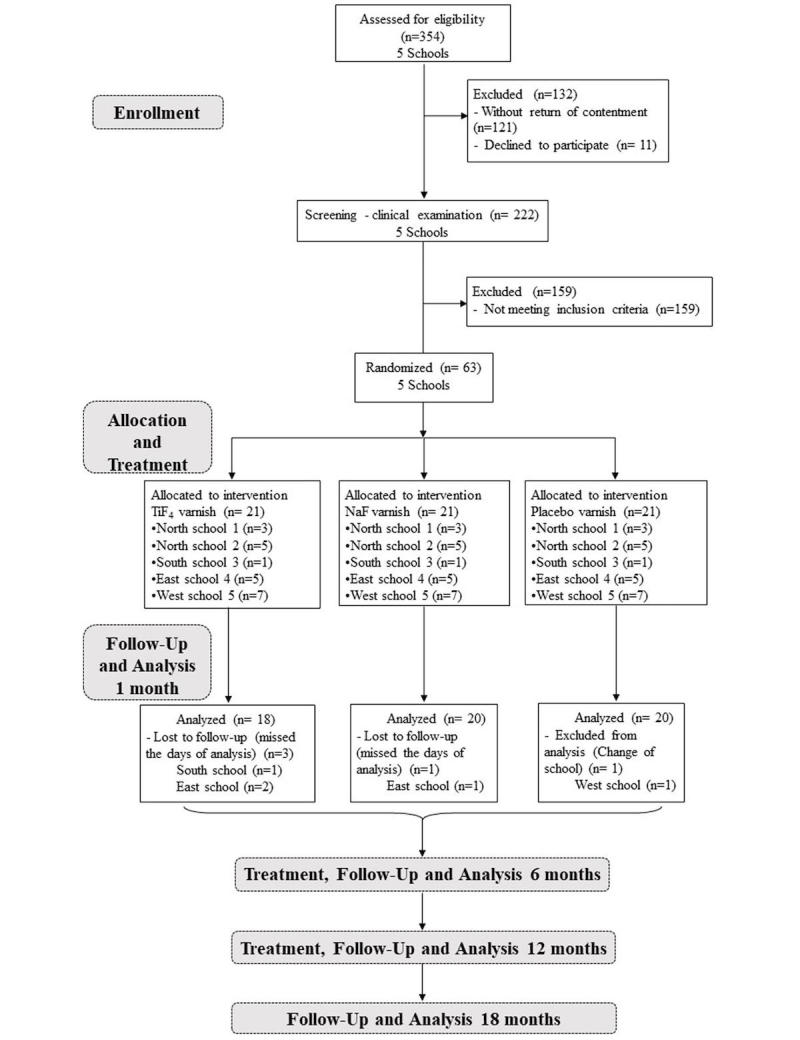
Flowchart of the study.

The treatment was conducted as described below. The teeth were submitted to clinical examination (using the International Caries Detection and Assessment System [ICDAS] and Nyvad indexes), and quantitative fluorescence changes were monitored by a quantitative light-induced fluorescence (QLF) device. The analyses were conducted after the first month and they will be carried out at the 6th, 12th, and 18th month of the study. Figure 1 summarizes the study protocol.

### Baseline Analysis

Two trained examiners (inter- and intraexaminer agreement, kappa>.8), not involved in the treatment application, are responsible for examining the children (NMS and BMS). The children were selected based on the analysis of smooth surfaces using the Nyvad index [12]. Only children aged 6-7 years, presenting at least 1 smooth surface with active carious lesions and the signed consents, were selected. The exclusion criteria were: children under orthodontic treatment; those who participated in another clinical study 3 months prior to the present study; those who underwent professional fluoride application 6 months prior to the present study; those under treatment with antibiotics or some other type of medicine (eg, patients with chronic diseases); or those with periodontal disease.

The distinction between active and inactive carious lesions was done through visual and tactile inspection. The active white spot lesions were defined as having a rough and opaque white surface [13]. All white spot lesions were further analyzed using QLF [14]. Furthermore, all permanent teeth surfaces were analyzed using ICDAS, which is a method of caries detection and evaluation that classifies the stages of the caries process [15]. The decayed, missing, filled teeth index (DMFT) was applied for the primary teeth (this data will be included in the regression analysis to check the influence of other variable on the results).

### Treatment

All children received instruction on cariogenic diets and oral hygiene, and were given supervised tooth brushing. The researchers provided new toothbrushes (Colgate Classic, Colgate-Palmolive), dental floss (Colgate, Colgate-Palmolive), and fluoride toothpastes (Colgate, 1450 ppm F^-^ as monofluoride phosphate [MFP], Colgate-Palmolive). The oral hygiene kit will be replaced every 3 months during the study.

The varnishes were applied on all permanent teeth once a week for the first 4 consecutive weeks [16] and they will be reapplied once at the 6th and 12th month of the study [17] by ASB. The application was done using a microbrush, under natural light, following the clinical steps:

1. Supervised tooth brushing by DMSS.
2. Relative isolation of teeth area with cotton rolls.
3. Drying of the teeth surfaces using sterile gauze.
4. Varnish application according to the manufacturer’s instructions.
5. Waiting for 5 minutes for solvent evaporation.
6. Removal of the cotton rolls.

The treatments were done during the afternoon. The children were instructed not to ingest liquid for 30 min, to have soft meals and to perform oral hygiene 4 hours after the application.

**Figure 2:**
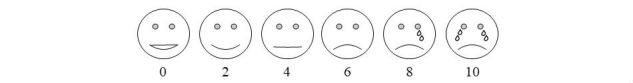
The Wong-Baker Visual Scale (WBPS), where 0 is very good (no pain/discomfort) and 10 is highly dissatisfied (worst possible pain/discomfort).

After each application, a visual scale (Figure 2) (Wong-Baker Pain Scale [WBPS]) was applied to assess the degree of patient satisfaction with the treatment. The scale is known to be one of the most effective tools for self-rated child pain [18]. The mean result of the first four varnish applications is described in the Results section.

### Clinical Examination

Clinical examinations were performed after dental hygiene activities, based on the clinical criteria for caries diagnosis outlined by Nyvad et al [19], which were modified as shown in Table 2 [12]. Only the smooth surfaces of all permanent teeth were considered and the clinical examination was carried out in an illuminated environment, using clinical probe and mirror, and sterile gauzes. In addition, the ICDAS index was used to assess all surfaces at baseline and will be applied again at the 18th month (Table 3).

The progression of white spot lesion occurs when the noncavitated lesion becomes a cavity (untreated cavity or restored tooth) or when a healthy surface is transformed into an active lesion (cavitated or not). The regression is defined as when the initial active white spot lesion is transformed into an inactive carious lesion or healthy surface. The data (degrees of freedom [df] = baseline Nyvad’s score – 1 month Nyvad’s score) was analyzed using a Kruskal-Wallis test.

**Table 2:** Modified Nyvad’s scores [12].

Score	Description
Score 0	Sound enamel
Score 1	Active white spot lesion – not cavitated
Score 2	Inactive white spot lesion – not cavitated
Score 3	Cavitated enamel (tooth with cavity, restored, or extracted)

**Table 3:** International Caries Detection and Assessment System (ICDAS) scores [15].

Score	Classification criteria
0	No or subtle change in enamel translucency after prolonged drying (5s) in area of biofilm accumulation
1	Visible white spot after drying (no loss of surface continuity) or pigmentation restricted to confines of a pit or fissure
2	White spot visible on wet surface (no loss of surface continuity) or pigmentation that extrapolates confines of a pit or fissure
3	Localized cavitation (or loss of continuity) in opaque or pigmented enamel
4	Underlying dark shadow from dentin, with or without cavitation of enamel
5	Cavitated enamel with exposure of the underlying dentin, involving up to half of the analyzed surface
6	Cavitated enamel with exposure of the underlying dentin, involving more than half of the analyzed surface

### Quantitative Light-Induced Fluorescence

QLF is applied to measure the changes in the enamel fluorescence of white spot lesions and to quantify its reversal or progression. A xenon arc lamp is used as a light source, and an optical filter system, producing blue light with a maximum wavelength of 370 nm, is connected to the microscope by a liquid light guide (Inspektor Research Systems BV, Amsterdam, The Netherlands). The fluorescence emitted by the tooth is collected with a charged coupled device (CCD)-video microcamera (Panasonic WV-KS 152, Matsushita Electric Industrial Co, Ltd, Osaka, Japan) equipped with high pass yellow filter (γ>520 nm) to exclude any excitation or ambient light that may reach the detector and a special dental mirror to reflect the image of the lesion connected to the camera [14].

After drying the tooth surface (for 5 s), images of clinically detected white spot lesions are obtained by QLF, in a completely dark environment. A computer program (Software Inspektor QLF 2.00f; Inspektor Research System BV, Amsterdam, The Netherlands) is utilized to display, store, browse, and analyze the images. The QLF parameters are: 1) the area of the lesion (white spot area [WS], mm^2^) that is the sum of all points within the lesion with fluorescence loss > 5%; and 2) the mean fluorescence loss (ΔF, %, detection threshold of 5%) [20].

The QLF analysis was performed at baseline and after 1 month. The differences between the 1 month and baseline values were calculated as follows: ΔWS area = WS area baseline – WS area after 1 month (the same for ΔΔF), where ΔWS is the variation of the white spot lesion area and ΔΔF is the variation of the mean fluorescence loss. The data were analyzed using the Kruskal-Wallis test. This analysis will be repeated after the 6th, 12th, and 18th months of the treatment by BMS.

### Statistical Analysis

The data will be subjected to statistical analysis using the GraphPad Instat and Prism (version 5.0 software) for Windows (GraphPad Software; San Diego, CA, USA). Firstly, the data will be checked for normality and homogeneity. A parametric or a similar nonparametric test will be applied to compare the treatments with respect to: 1) the prevention of new carious lesions (ICDAS and Nyvad indexes); 2) regression or progression of previous active white spot lesions using the Nyvad index; 3) regression or progression (gain or loss of fluorescence, respectively) of previously active white spot lesions using QLF (at 0, 1, 6, 12 and 18 months).

## Results

This protocol refers to an ongoing clinical study funded by the São Paulo Research Foundation (FAPESP-2015/14149-1) and the National Council for Scientific and Technological Development (CNPq-401313/2016-6).

Treatment, Clinical Examination and Quantitative Light-Induced Fluorescence

Figure 1 shows the number of children by school enrolled in the research so far. All enrolled children (n=63) were underwent 4 weeks of treatment and almost all of them (n=58) were analyzed after 1 month. Five out of 63 children (8%) dropped out after the first month of analysis (n total=58). No significant differences in caries prevention, regression or progression were found among the treatments at the first month (Tables 4 and 5). The degree of patient satisfaction with the treatment after the varnish applications is displayed in Tables 6 and 7.

**Table 4:** Nyvad’s scores [12] at the baseline and after 1 month of treatment (final) for TiF_4_, NaF and placebo treatments.

Measures	TiF_4_			NaF			Placebo	
	Baseline	Final	Baseline		Final	Baseline		Final
Nyvad index, mean±SD	1.0±0.00	1.0±0.00	0.97±0.15		1.03±0.15^a^	0.99±0.06		1.08±0.24^b^
Df^c^, median (min-max)	0.0 (0.0 to 0.0)			0.0 (–0.7 to 0.0)			0.0 (–1.0 to 0.0)	

^a^One patient presented two teeth that progressed from score 0 to 1 and 1 patient had one lesion that progressed from score 1 to 3.

^b^One patient presented one tooth that progressed from score 0 to 3 and one patient had one lesion that progressed from score 1 to 3.

^c^Df: degrees of freedom. Df = baseline – final value, where positive values indicate regression and negative values indicate lesions progression. Kruskal-Wallis Test (*p*=.39).

**Table 5:** Median (minimum-maximum values) obtained in quantitative light-induced fluorescence (QLF) analysis at the first month compared to the baseline.

Type of varnish	ΔWS area (mm^2^)^a,b^	ΔΔF (%)^b,c^
TiF_4_	0.01 (–9.15 to 1.19)	–1.29 (–16.30 to 4.74)
NaF	0.17 (–2.38 to 1.47)	–0.55 (–5.80 to 6.10)
Placebo	0.19 (–1.14 to 4.36)	–0.23 (–5.17 to 5.10)

^a^ΔWS: variation of the white spot lesion area. For ΔWS, negative values mean progression (demineralization), and positive values, regression (remineralization). The opposite is valid for ΔΔF.

^b^Kruskal-Wallis Test (*p*=.59 and *p*=.45, respectively).

^c^ΔΔF: variation of the mean fluorescence loss.

**Table 6:** Mean percentage (%) of the degree of patient satisfaction after the first 4 applications of the TiF_4_, NaF and placebo varnishes using the Wong-Baker Pain Scale.

Wong-Baker Pain Scale	Type of varnish, mean±SD (%)		
	TiF_4_	NaF	Placebo
0	75±8	86±6	76±11
2	20±5	12±6	17±9
4	5±4	2±3	3±3
6	0±0	0±0	3±3
8	0±0	0±0	0±0
10	0±0	0±0	1±3

**Table 7:** Number of patients included in the analysis of satisfaction (Wong-Baker Pain scale) after the first four varnish applications.

Wong-Baker Pain Scale	Number of patients by varnish type											
	TiF_4_				NaF				Placebo			
	1st^a^	2nd	3rd	4th	1st	2nd	3rd	4th	1st	2nd	3rd	4th
0	18	15	16	14	19	17	19	17	18	16	13	14
2	3	5	4	5	1	3	2	4	1	4	5	4
4	0	1	1	2	1	1	0	0	1	0	0	1
6	0	0	0	0	0	0	0	0	0	0	1	1
8	0	0	0	0	0	0	0	0	0	0	0	0
10	0	0	0	0	0	0	0	0	0	0	1	0
Total	21	21	21	21	21	21	21	21	20	20	20	20

^a^The first (1st), the second (2nd), the third (3rd) and the fourth (4th) varnish application.

We expect to confirm the efficacy of the TiF_4_ varnish compared to one based on NaF for the prevention and treatment of carious lesions at the end of the present study (18 months) as we have previously found under in vitro and in situ protocols.

## Discussion

Previous systematic reviews have shown no significant differences between the anticaries performances of fluoride (mainly NaF) included in different products such as gel, varnish and toothpaste [1,2]. However, varnish has some advantages over the other products, since it adheres to the tooth surface allowing for a long time of contact between the fluoride and teeth. It also presents low systemic toxicity, and is well tolerated and accepted by patients, especially children [1,2,21]. Therefore, we tested the anticaries effect of an experimental TiF_4_ varnish.

Incorporating TiF_4_ into a varnish allows for a longer contact time with enamel, thereby improving the reaction of titanium with the tooth apatite and facilitating the formation of a glaze-like layer on the tooth surface that is rich in titanium dioxide, hydrated titanium phosphate and CaF_2_[6,10]. Due to its low pH, a TiF_4_ varnish is able to enhance the enamel fluoride uptake compared to a NaF varnish [10]. The varnish may also reduce TiF_4_ contact with soft tissues compared to a rinse solution, reducing the possibility of cytotoxicity due to its low pH [22]. A recent study has shown that a TiF_4_ varnish presents similar levels of toxicity to murine fibroblast lineage (NIH/3T3) cells as a NaF varnish [22]. To check for possible side effects of the TiF_4_ varnish, patient satisfaction was evaluated using a simple, but effective tool for self-rated child pain [18].

Previous studies have shown that the application of fluoride varnish once a week for 4 consecutive weeks (4 applications in a one-month interval) has been effective in accelerating remineralization of white spot lesions [16]. On the other hand, biannual applications are effective for the prevention of new carious lesions [17,21]. The focus of the research is preventing and treating carious lesions on smooth tooth surfaces, which is where fluoride varnish is predominantly used [1]. For occlusal surfaces, other treatments are often indicated, such as fissure sealants, despite a recent systematic review has demonstrated positive results using NaF varnish on occlusal surfaces [23].

The most common method for detecting caries is the visual-tactile technique by using the ICDAS, Nyvad and DMFT indexes. However, other noninvasive techniques for the detection of early carious lesions have been developed, such as QLF and DIAGNOdent, which are especially used for research purposes [24]. The traditional DMFT index is based on detecting carious lesions at the cavitated level only, but it is not a good method for identifying these lesions at a very early stage [25]. On the other hand, ICDAS is an accurate and reproducible method for discovering early lesions in enamel and also for detecting changes over time [24]. Braga et al [26] compared two methods of visual inspection (ie, Nyvad and ICDAS), and both presented good reproducibility and validity in regards to identify and estimate lesion depth, which justifies their inclusion in the present study.

The QLF is a sensible quantitative clinical method with good repeatability and reproducibility, requiring a smaller number of participants (that may decrease the impact of dropout for longitudinal studies) compared to the visual analysis [14]. The QLF is able to quantify small mineral changes that might not be detectable in the visual inspection. However, the method is not reliable for detecting the portion of the subsurface lesion where minerals are gained or lost [14,20]. Therefore, we combined the visual inspection with a complementary method (QLF) to better detect and quantify carious lesions at a very early stage [24].

Despite our hypothesis on the effectiveness of the TiF_4_ varnish, results obtained at the first month of this study demonstrate that the differences between TiF_4_ and NaF did not reach significance, due to the slight changes in the visual analysis and in the fluorescence loss. If the TiF_4_ varnish proves to be better at controlling dental caries compared to the NaF version by the end of our study, it shall be marketed and distributed at the individual level and to community programs, in order to prevent and treat dental carious lesions in children in the future.

## Abbreviations

CaF_2_: calcium fluoride
CCD: charged coupled device
CNPq: National Council for Scientific and Technological Development
DMFT: decayed, missing, filled teeth index
DIAGNOdent: Brazilian device used for measuring teeth demineralization in vivo.
EMEF: municipal school of fundamental education
F: fluoride
FAPESP: The São Paulo State Research Foundation
ICDAS: International Caries Detection and Assessment System
MFP: monofluoride phosphate
NaF: sodium fluoride
NIH/3T3: murine fibroblast lineage
QLF: quantitative light-induced fluorescence
SnF_2_: stannous fluoride
TiF_4_: titanium tetrafluoride
WBPS: Wong-Baker Pain Scale
WS: white spot area
ΔF: mean fluorescence loss
ΔWS: variation of the white spot lesion area
ΔΔF: variation of the mean fluorescence loss
